# Information from social ties predicts conspiracy beliefs: Evidence from the attempted assassination of Donald Trump

**DOI:** 10.1093/pnasnexus/pgaf193

**Published:** 2025-06-14

**Authors:** Katherine Ognyanova, James N Druckman, Jonathan Schulman, Matthew A Baum, Roy H Perlis, David Lazer

**Affiliations:** School of Communication and Information, Rutgers University, 4 Huntington St., New Brunswick, NJ 08901, USA; Department of Political Science, University of Rochester, 333 Harkness Hall, Rochester, NY 14627, USA; Institute for the Study of Citizens and Politics, University of Pennsylvania, 133 S. 36th St., Philadelphia, PA 19104, USA; Harvard Kennedy School, Harvard University, 79 John F. Kennedy St., Cambridge, MA 02138, USA; Department of Psychiatry, Harvard Medical School, 25 Shattuck St., Boston, MA 02114, USA; Network Science Institute, Northeastern University, 177 Huntington Ave., Boston, MA 02148, USA

**Keywords:** conspiracy beliefs, social ties, social media, partisanship, misinformation

## Abstract

Belief in conspiracy theories has significant social and political consequences. While prior research has focused primarily on psychological predispositions as drivers of conspiracy beliefs, relatively less is known about the role of social networks. Here, we examine how information received from different sources is linked to the endorsement of conspiracy theories, using the 2024 attempted assassination of presidential candidate Donald Trump as a case study. In surveys conducted days after the attack, social media was the most commonly reported source of conspiracy theories about the event. At the same time, information consumption on social media was not consistently associated with stronger conspiracy beliefs. In contrast, information received through interpersonal ties was more closely linked to belief in both left-leaning and right-leaning conspiratorial narratives. These findings highlight the importance of examining the social dimensions of conspiracy belief formation. Understanding how interpersonal communication shapes conspiracy beliefs is critical for explaining their spread and persistence. Future research would benefit from further investigating the social contexts that sustain conspiratorial thinking.

Significance Statement Belief in conspiracy theories has major social and political consequences. While prior research emphasizes psychological factors, less is known about how social networks shape conspiracy beliefs. We examine this question using the 2024 attempted assassination of presidential candidate Donald Trump as a case study. Surveys conducted days after the attack show that social media was the most commonly reported source of conspiracy theories. However, consuming information on social media was not consistently associated with stronger conspiracy beliefs. In contrast, information received through personal networks was more closely linked to endorsement of both left- and right-leaning conspiracy narratives. Our findings highlight the importance of examining the social dimensions of conspiracy belief formation.

## Introduction

Conspiracy theories have significant social and political consequences, influencing public trust, political behavior, and, in rare cases, inciting violent action. While previous research has largely focused on individual psychological predispositions that foster belief in conspiracies, the role of interpersonal communication and information transmission has received relatively less attention. Understanding how social contexts shape and reinforce conspiracy beliefs is crucial for explaining their spread and adoption.

In this study, we investigate how information sources are linked to belief in newly emerging conspiracy theories. To explore these dynamics, we use the 2024 attempted assassination of presidential candidate Donald Trump as a case study.

On 2024 July 13, a 20-year-old man from Bethel Park, Pennsylvania, attempted to assassinate Donald Trump at a presidential campaign rally. The shooting created conditions ripe for the emergence, spread, and acceptance of conspiracy theories: it was an unusual and high-profile event with unseen origins that potentially pit good against evil (1). As with John F. Kennedy's assassination, its high profile paved the way for a range of implausible explanations (2).

Almost immediately after the event, conspiracy theories—that is, attempts to explain the causes of events through secret plots by powerful actors (3)—emerged on both sides of the political spectrum. On the right, one popular conspiracy theory claimed that the shooting was arranged by Democratic operatives aiming to prevent Trump from winning the 2024 election. A social media post about the event from Representative Mike Collins stated, “Joe Biden sent the orders.”^a^ Others took this theory even further, with radio host Alex Jones claiming that the attack was a part of a failed deep state coup.^b^ On the political left, another narrative suggested that the assassination attempt was staged by Republican political actors to boost the popularity of their preferred candidate. This statement was often accompanied by a misleading photograph of Donald Trump (taken in 2022 rather than 2024), showing no damage to his ear, which was injured in the incident.^c^

Scholars have documented various psychological drivers of conspiracy beliefs, including motivated reasoning and conspiratorial thinking (4–6). The role of communicative social factors, however, has been studied much less. In a recent review, Douglas and Sutton (7) claim that “the communicative aspects of conspiracy theories have been largely ignored… theories of conspiracy belief may overemphasize individual-level factors” (p. 286).

We examine the role of information sources and social dynamics in people's exposure to and acceptance of conspiracy theories about the Trump assassination attempt. The high-profile, singular nature of this event enabled us to study the link between social networks and conspiracy beliefs. This incident resulted in a rapid information spread, distinct from the diffusion of other conspiracy ideas evolving over a longer time period.^d^ Communication processes are harder to credibly capture in cases when conspiracy theories get modified over time by many individuals. Considerable measurement errors may arise from poor recall of self-reported information consumption going back months or years. Recall about a single prominent event that happened mere days ago would likely be considerably more reliable (8,9). Furthermore, interpersonal networks are likely to play a key role—people depend on those around them for political information (10–12), and these discussions can moderate other sources such as media (13).

## Results

To study conspiracy theory exposure and beliefs, we conducted a survey from 2024 July 17 to 21. The vast majority of our respondents (95%) were aware of the July 13 Trump assassination attempt. Most of those who knew about the event reported getting information about it from television (64%), followed by social media (43%) with fewer people reporting reliance on interpersonal social networks (30%).

**Fig. 1.**
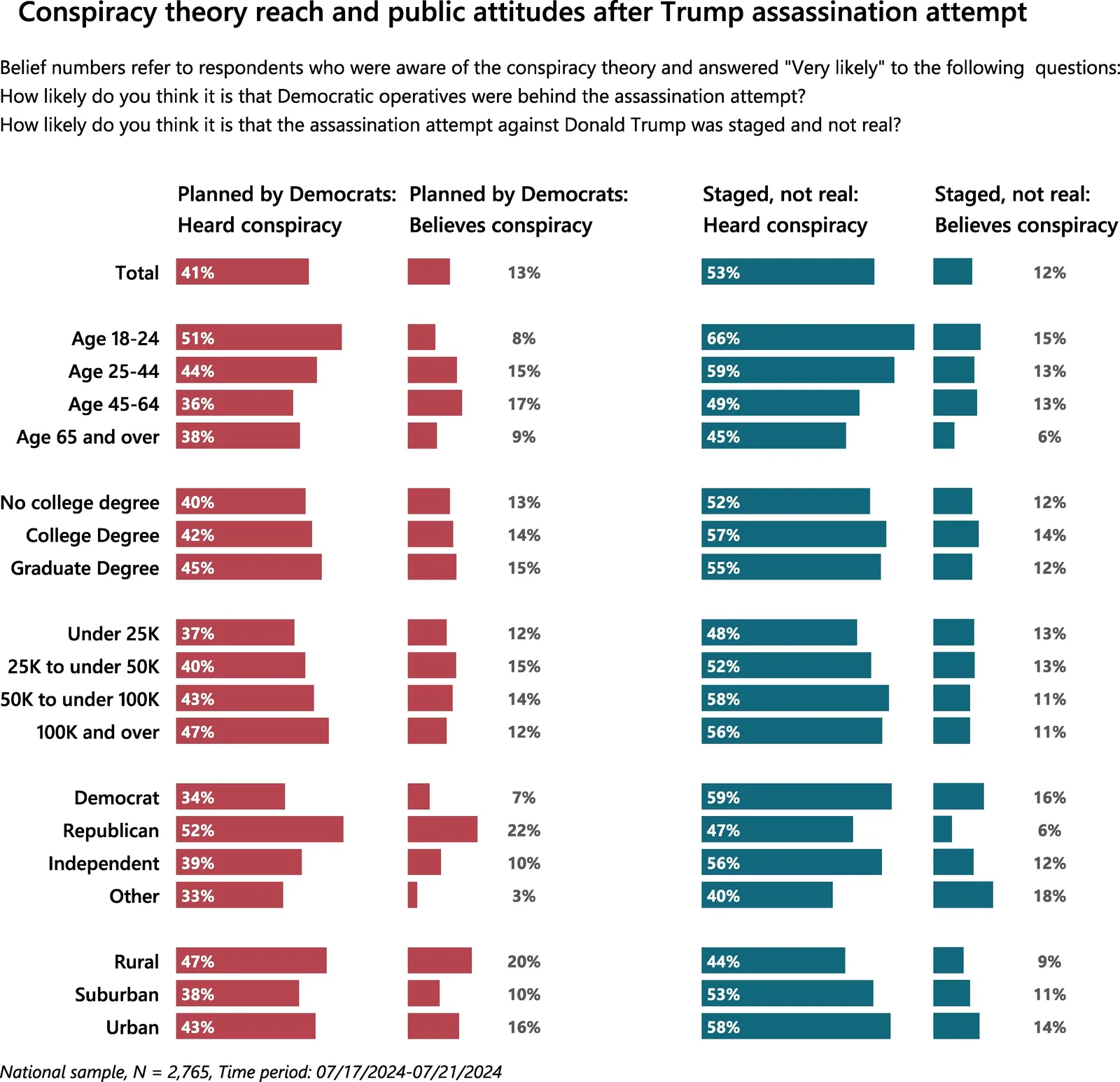
Percent respondents who reported awareness and belief in the conspiracy theories emerging after the attempted assassination of Donald Trump.

**Fig. 2.**
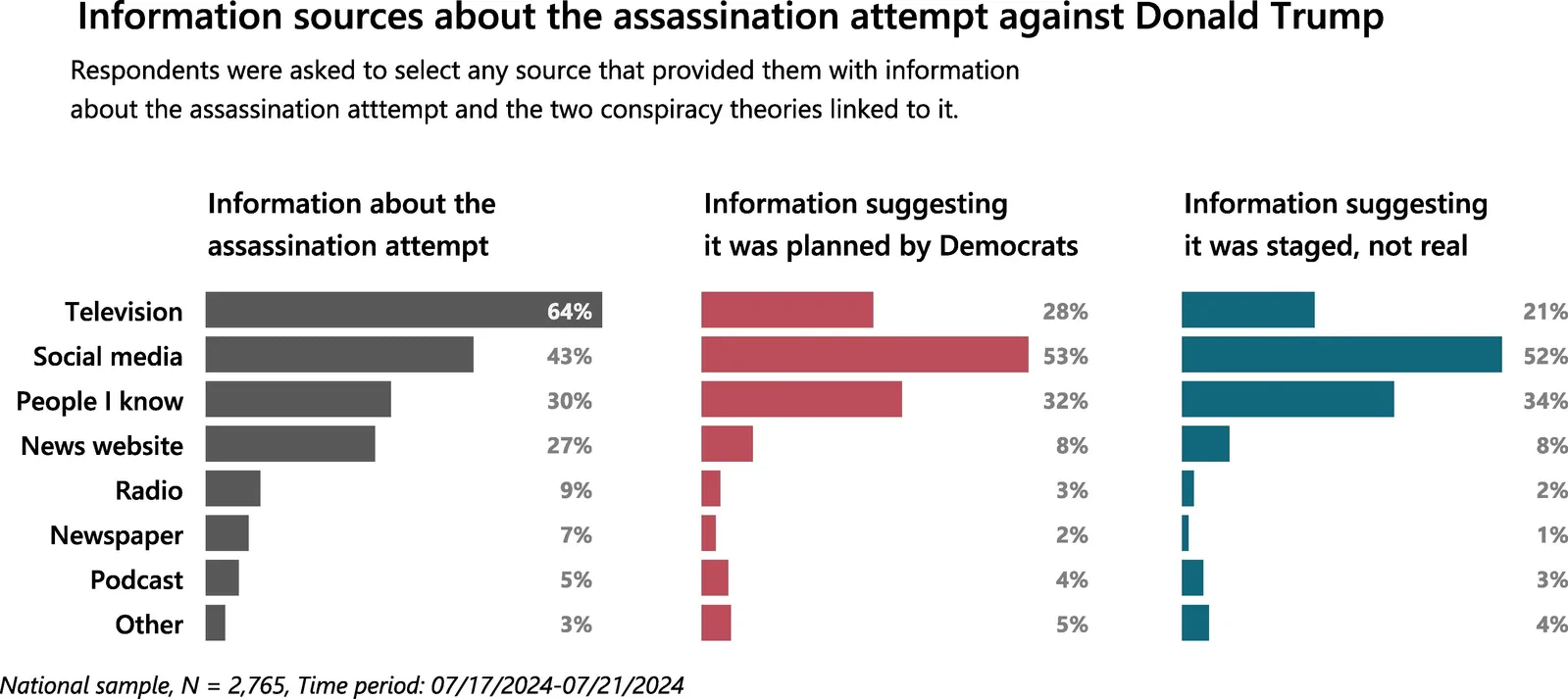
Sources of information about the assassination attempt against Donald Trump.

A considerable proportion of the respondents were also aware of the rumors about the event (Fig. 1). A total of 41% had heard the right-leaning conspiracy theory that the shooting was planned by Democratic operatives. Of those individuals, 53% said they found this information on social media, 28% saw it on television, and 32% reported hearing it from interpersonal social networks (see Fig. 2 and Supporting Information).^e^ Close to a third of respondents who heard this rumor believed it was “very” (13%) or “somewhat” (16%) likely to be true.

Over half of the respondents (53%) were aware of the left-leaning conspiracy theory that the attempted assassination was staged and not real. Of those, 52% heard this idea on social media, 34% learned it from interpersonal social networks, and 21% saw it on television (see Fig. 2 and Supporting Information). Close to a third of respondents who had heard the rumor thought it might be true (12% said it was “very likely” and 17% said it was “somewhat likely”). Consistent with previous research (14), social media was a primary source linked with exposure to both conspiracy theories.

**Fig. 3.**
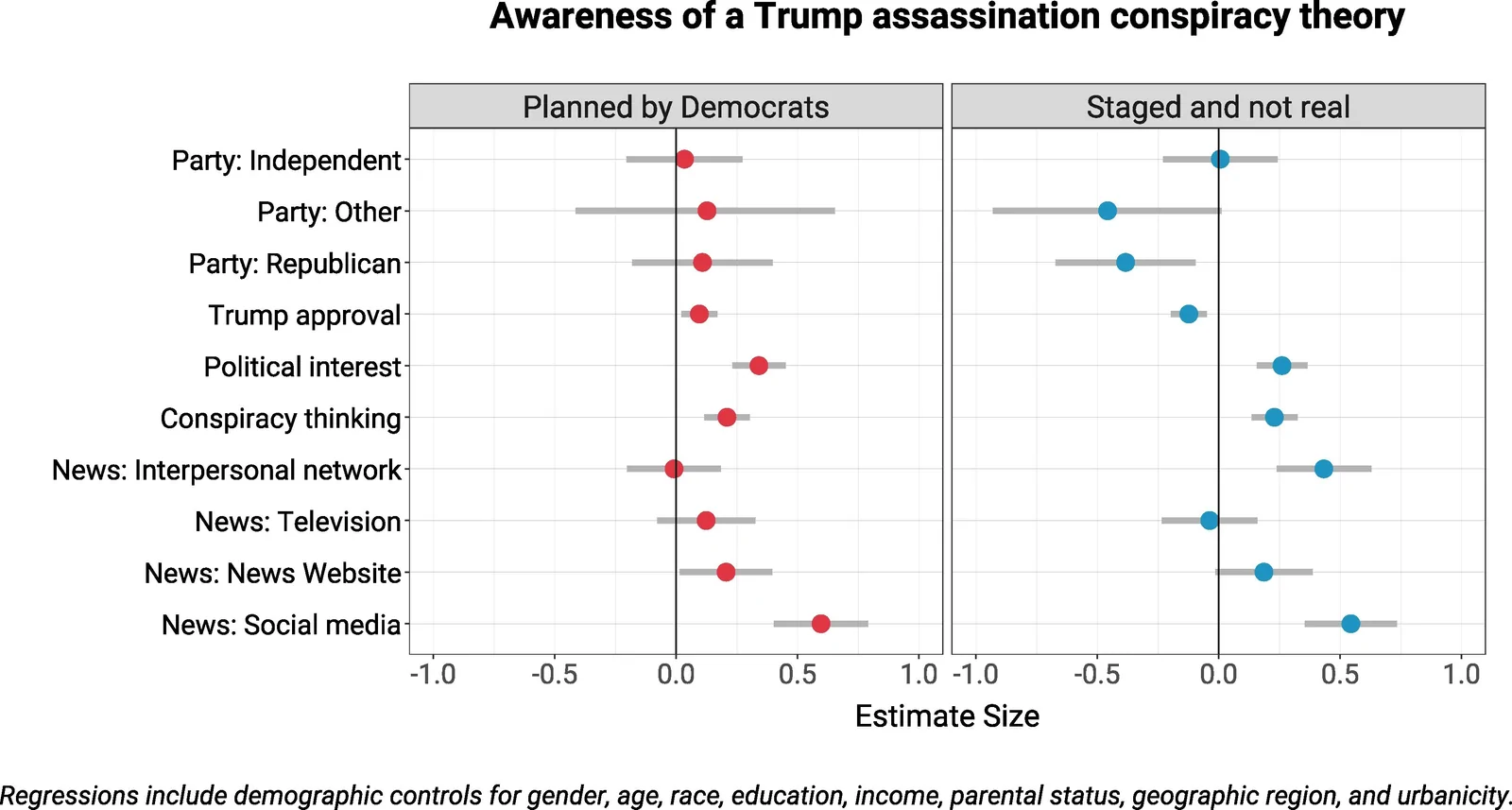
Awareness of conspiracy theories about the assassination attempt against Donald Trump. Regressions include demographic controls for gender, age, race, education, income, parental status, geographic region, and urbanicity.

We used logistic regression to examine the likelihood of encountering each conspiracy theory. Across both conspiracies (see Fig. 3 and Tables S1 and S2), we found that conspiratorial thinking disposition, political interest, Trump approval, and consuming information about the assassination on social media significantly correlated with self-reported exposure. No other news source was consistently correlated with exposure to both conspiracies.

**Fig. 4.**
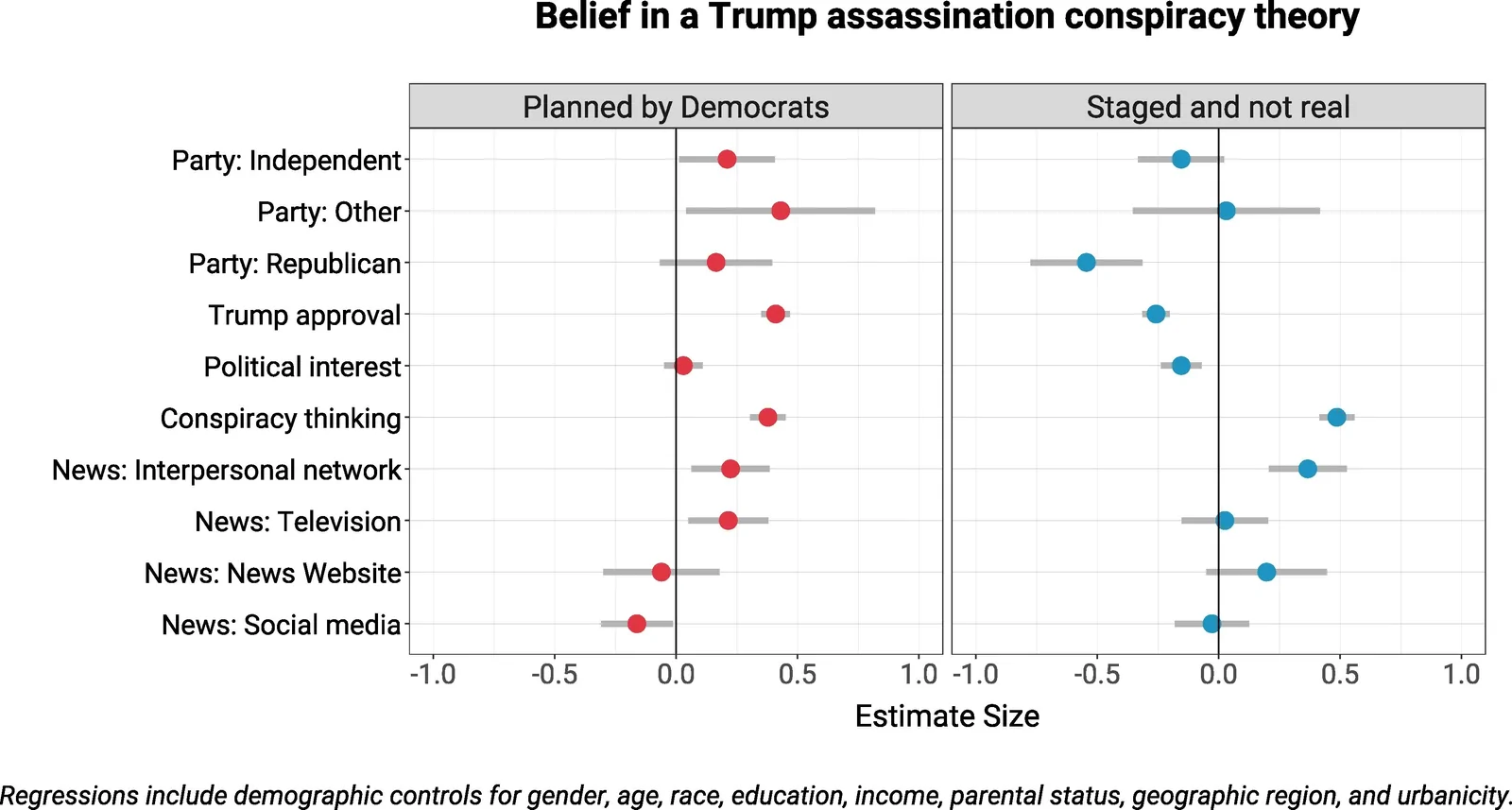
Belief in conspiracy theories about the assassination attempt against Donald Trump. Regressions include demographic controls for gender, age, race, education, income, parental status, geographic region, and urbanicity.

Interpersonal social network sources were significantly and positively correlated with hearing the conspiracy that the assassination attempt was staged and not real, but not with the right-leaning Democratic operative conspiracy. This offers some evidence of an association between networks and conspiracy spread, although as noted in previous works, social media has a stronger relationship (4). Separate OLS regressions examined predictors of believing the conspiracy theories among those who had heard about them (Fig. 4 and Tables S3 to S6). Interpersonal social networks were the only information source consistently and positively related to holding both conspiracy beliefs. Substantively, hearing about a conspiracy from interpersonal ties was associated with about 0.2–0.4 increase in the perceived likelihood that the theory was true on a 0–4 scale (although we urge caution in generalizing precise point estimates). We further found that conspiratorial thinking, partisanship, and Trump approval were significantly associated with belief in the expected directions. Contrary to the exposure results, hearing about a conspiracy theory from social media was not positively related to believing it.

## Discussion

The interpersonal aspects of conspiracy beliefs have received little attention in previous academic works. This gap in the literature reflects the difficulty of studying communication that likely occurs sporadically and inconsistently over time. In contrast, the enduring influence of psychological dispositions can be easier to capture. In this work, we leveraged a salient national event that generated multiple conspiracies to document the relationship between conspiracy beliefs and interpersonal networks. As suggested by persuasion research, information delivered by social ties is associated with higher probability of belief. The result affirms Douglas and Sutton's assertation that conspiracy theories are “inherently social not only in their content but also in their purpose: They are beliefs that people share in the hope of achieving social goals… it is deeply misleading to characterize conspiracy theories merely as beliefs that individuals hold” (7, p. 286).

Our hope is that these results will spur more work on the social aspects of conspiracy beliefs, including their causal relations. Here, we have avoided making causal claims, as it is possible that people select social ties for reasons that are not independent of their propensity to believe conspiracies. Future work would benefit from exploring not only belief in a conspiracy theory, but also the certainty or confidence with which that belief is held (15, 16). Scholarship in this area should also further explore the nature and impact of conspiracy-driven social networks (17).

Another obvious question is whether our findings generalize to different scenarios and how interpersonal social networks interact with other variables, such as actual social media exposure and consumption. It is intriguing that in our data, self-reported social media consumption is correlated with exposure, but not conspiracy beliefs. The main takeaway is that conspiracy beliefs are a social phenomenon and studying them as such will enhance our understanding of their origins and consequences.

## Materials and methods

The data were collected via an online nonprobability sample with quotas for gender, race, age, and region. Respondents (*n* = 2,765) were recruited by the panel sample vendor PureSpectrum between 2024 July 17 and 21. The research was reviewed and approved by the Institutional Review Board at Rochester University (IRB STU00219054). Informed consent was obtained through an online form presented to all participants.

To improve the representativeness of the sample relative to the US population, we generated poststratification weights based on US Census Bureau data for demographics including race/ethnicity, age, gender, education, and geographic region. We used NCHS urban–rural classification data for urbanicity. We also included interlocking gender-by-age-by-race categories, as well as education-by-age and education-by-race. Finally, we adjusted the weights based on Federal Election Comission (FEC) data on turnout and vote choice in the 2020 presidential election. Additional details about the data, its collection, and quality checks are provided in Section 1 of the supporting information.

We measured self-reported information consumption by asking people who knew of the Trump assassination attempt where they obtained information about it. Respondents selected all applicable answers from eight options including *people I know*, *television*, *social media*, and *news websites*. The “social media” option captured a communication channel that could carry multiple types of content, including posts from social ties, strangers, or news media. Since respondents could choose all relevant options, we were able to isolate the interpersonal and media sources by including those as separate binary variables in the model. While self-reported consumption measures are certainly imperfect, extant work suggests that the specificity of the topic and recency of the event enhance accuracy (8, 9).

To measure belief in each of the two conspiracy theories, we asked respondents how likely or unlikely they thought it was to be true. While asking about likelihood does not eliminate potential acquiescence bias, it does avoid the most obvious problems stemming from use of agree–disagree answer formats. This measure does not directly record one's certainty or confidence in their belief (15, 16). Future work would benefit from further exploring belief strength.

We conducted separate logistic regressions to examine exposure to the two conspiracy theories, and OLS regressions to examine correlates of believing the theories. Belief was measured on a 0–4 scale ranging from reporting it “very unlikely” to “very likely” that the conspiracy was true. The belief regressions were based on the subsample of respondents who had heard each theory (*n* = 1,081 for the right-leaning and *n* = 1,348 for the left-leaning one). Variables included gender, race/ethnicity, age, education, income, parental status, region, urbanicity, political party, ideology, approval of Donald Trump, interest in political news, conspiratorial thinking, and sources of information.

See Supporting Information for survey item text, descriptive statistics, regression results, and detailed discussion of our sample and measures.

## Supplementary Material

pgaf193_Supplementary_Data

## Data Availability

Replication materials (18) for this paper are available at doi.org/10.17605/OSF.IO/5RHTN.

## Notes

a
https://twitter.com/MikeCollinsGA/status/1812257581655531669

b
https://www.cbsnews.com/news/trump-rally-shooting-misinformation-conspiracy-theories/

c
https://apnews.com/article/trump-rally-shooting-fact-focus-election-c02bcff9f4210e890c0003a1f85044f7

d For instance, conspiracy theories about climate change, vaccinations, or government control.

e Numbers sum to more than 100% as respondents could select multiple sources where they encountered the information.

## Preprints

An early version of this manuscript was posted as a preprint: doi.org/10.31219/osf.io/hfbeu.
