# Correction to: Information from social ties predicts conspiracy beliefs: Evidence from the attempted assassination of Donald Trump

**DOI:** 10.1093/pnasnexus/pgag298

**Published:** 2026-09-08

This is a correction to: Katherine Ognyanova, James N. Druckman, Jonathan Schulman, Matthew A. Baum, Roy H. Perlis, David Lazer, Information from social ties predicts conspiracy beliefs: Evidence from the attempted assassination of Donald Trump, *PNAS Nexus*, Volume 4, Issue 6, June 2025, pgaf193, https://doi.org/10.1093/pnasnexus/pgaf193

The following competing interest disclosure has been added: Editor David G. Rand has received research funding from Google and Meta. Editor David G. Rand has coauthored with James N. Druckman.

