# Supplementary material for: Information from social ties predicts conspiracy beliefs: Evidence from the attempted assassination of Donald Trump
Source: PNAS Nexus. 2025 Jun 14;4(6):pgaf193. doi: 10.1093/pnasnexus/pgaf193 (PMC12203515; doi:10.1093/pnasnexus/pgaf193)
Supplement: pgaf193_Supplementary_Data [file pgaf193_supplementary_data.pdf]

Supporting Information

Ognyanova, K., Druckman, J. N., Schulman, J., Baum, M. A., Perlis, R. H., & Lazer, D. (2025). Information from Social Ties Predicts Conspiracy Beliefs: Evidence from the Attempted Assassination of Donald Trump. *PNAS Nexus*.

Contents:

1. **Methodological Considerations**
2. **Sample Description**
3. **Question Text**
4. **Descriptive Information**
5. **Regression Results**
6. **Supporting Information References**

1. Methodological Considerations

There are four methodological considerations that we discuss here: the sample, our conspiracy belief measures, our information measures, and causal inference.

Sample

The historic gold standard in survey research involves drawing a probability sample of the population. In this case, every member of the population has a known, non-zero chance of being in the sample. A non-probability survey is one where there is no random selection. While some evidence suggests that estimates from non-probability samples are not as accurate as those from probability samples (MacInnis et al. 2018, Bradley et al. 2021, Mercer and Lau 2023), others show that non-probability samples can offer accurate estimates (Gelman et al. 2016, Enns and Rothschild 2021, Holliday et al. 2021).

Our survey comes from the Civic Health and institutions Project (CHIP50). It relies on a non-probability sample. This does not automatically undermine our ability to draw inferences: the American Association for Public Opinion Research (AAPOR)'s Task Force Report on non-probability sampling states "Researchers and other data users may find it useful to think of the different nonprobability sample approaches as falling on a continuum of expected accuracy of the estimates" (Baker et al. 2013). The report also states that "non-probability sampling is a collection of methods rather than a single method, and it is difficult if not impossible to ascribe properties that apply to all non-probability sampling methodologies."

It is thus misleading to make general blanket statements about "non-probability surveys." In our case, survey participants are recruited by 22 vendors who use a variety of strategies and incentives to maintain online respondent panels. Respondents are channeled to surveys through PureSpectrum, an online polling platform that works with panel providers from all 50 states and D.C. PureSpectrum provides initial respondent identification, deduplication, and screening for quality, demographic qualifications, and location. Using the PureSpectrum API, the CHIP50 team has developed a system to launch and monitor multiple survey projects in all states, based on pre-specified quotas and qualifications (see below). The CHIP50 team monitors recruitment in real time.

For this paper, we relied on a national sample (more details are included in the next section). We used sampling quotas, as well as post-stratification weights (a prevalent practice for both non-probability and probability samples). These efforts increase the representativeness of a sample relative to the population when it comes to the measured variables (Vehovar et al. 2016). For sample quotas, we used gender (Male, Female), age (18-24, 25-34, 35-44, 45-54, 55-64, 65-99), race (Black, Asian, Hispanic, White, Other), and census region. Each quota is determined by the corresponding 2020 U.S. Census Bureau data. To generate post-stratification weights, we used U.S. Census Bureau data for population demographics including race/ethnicity, age, gender, education, and geographic region. We use NCHS urban-rural classification data for urbanicity. We also included interlocking gender-by-age-by-race categories, as well as education-by-age and education-by-race.

CHIP50 surveys employ several methods to improve measurement. We include a CAPTCHA test to prevent bots from taking the survey. To enhance response validity, CHIP50 attempts to identify and remove bogus and problematic respondents. We exclude anyone who fails one of two basic attention checks that straightforwardly ask participants to choose a specific response option. The remaining respondents are evaluated based on their performance on a series of quality checks, including: (1) duplicate responses, (2) prevalence of item non-response, (3) short survey completion time, (4) straight-lining consecutive item responses (5) selecting a large number of religions (6) reporting a household with over 20 family members (7) identifying as a “Democrat” and “very conservative”; or a “Republican” and “very liberal”, and (8) giving a meaningless answer to an open-ended question requesting a state name. Respondents who fail multiple quality checks are removed from the data. This additional filtering affects a relatively small percentage of respondents who completed the survey, with the majority disqualified for failing attention checks. In the survey used for this study, 15% of respondents were removed.

The CHIP50 project has undertaken extensive validation efforts to assess the accuracy of estimates from CHIP50 surveys.

Our validation work finds that:

1) Estimates closely match COVID-19 vaccination rates produced by the CDC (Green et al. 2023), and a major national probability survey (Quintana-Mathe et al. 2024).

2) Estimates closely match COVID-19 infection rates as tracked by Johns Hopkins University relying on CDC state-level estimations (Quintana-Mathe et al. 2024), and wastewater estimates (Santillana et al. 2024).

3) Estimates closely match the two-party vote share in the 2020 elections as estimated by major election polls (Radford et al. 2022).

4) Estimates closely match other surveys or administrative data across domains including BLM protests (Simonson et al. 2024), experiencing symptoms of depression (Baum et al. 2024), and gun purchasing (Lacombe et al. 2022).

A key feature of the data collection is that the project is able to launch a survey very quickly. That ability was crucial in this case, given the quickly unfolding nature of the event.

Measuring Conspiracy Beliefs

Douglas and Sutton (2023: 273) explain that “Belief in conspiracy theories—like many other psychological constructs—was and is typically measured using self-report scales. In such scales, participants are asked to rate their disagreement/agreement on a Likert-type scale with each of several specific… or general… statements.”

This is the approach we take here. For each of the two conspiracy theories, we ask how likely or unlikely respondents believe it is to be true. While asking about likelihood does not eliminate potential acquiescence bias, it does avoid the most straightforward incarnation of the bias (i.e., agree-disagree answer formats). As mentioned in the report text, the measure also does not gauge one’s certainty or confidence in their belief (Berinsky 2023, Graham 2023); future work would benefit from doing so.

Measuring Information Consumption

We measure the source of information about the conspiracies by asking respondents where they received the information. We offer a set of options, from which they could choose more than one: people I know, television, radio, newspaper, news website, social media, podcast, and other. Our construct is information consumption. This differs from mere exposure which is most directly captured by digital trace data. While using more objective trace data comes with obvious advantages, the downsides are that it does not ensure consumption (Conrad et al. 2021, Konitzer et al. 2021) and it is often difficult to identify the precise content users see.

(For more general discussion on digital trace data, see Jungherr 2018, Bosch and Revilla 2022.)

We recognize that self-reported information consumption measures are susceptible to mis-reporting (e.g., Scharkow 2016). As mentioned in the text, two aspects of our measures provide confidence, however. First, our acute topical focus (rather than general focus in “conspiracies” or “politics”) increases the accuracy of the measures. Guess et al. (2019: 254) point out that with specific content, “The good news is that self-reports are correlated with observed behavior… self-reports of social media use are meaningful; they are (perhaps surprisingly) accurate and correlated with our objective measure…” (also see Dilliplane et al. 2013, Scharkow 2016, Settle 2018, Barthel et al. 2020). Second, by asking about consumption of very recent information (i.e., the recently developed conspiracies), we reduce the cognitive load on respondents and minimize the likelihood and extent of over-reporting (Tourangeau et al. 2000).

The measure is of course not perfect. The most notable threat, notwithstanding our prior comments, concerns accuracy and the possibility of respondents’ mis-reporting consumption (e.g., Konitzer et al. 2021, Parry et al. 2021). That said, this is less of a challenge here since we are not interested in explicit comparisons between information sources. When one compares sources explicitly, an obvious question is whether one of the measures has more error than the other. Instead, our focus is on documenting relationships, which means we rely less on assumptions that different information sources have analogous measurement properties.

Causation

There is an inherent tradeoff in studying the relationships between information sources and beliefs. When the goal is to make causal statements, experimentation is unrivaled as a method (Druckman 2022). Indeed, it has become a dominant approach in studying social media effects (e.g. Nyhan et al. 2023). We did not employ an experiment for two reasons. First, we leveraged the opportunity to study a fast-breaking conspiratorial reaction to the Trump assassination attempt. This required immediate fielding, and it would have been difficult, if not impossible, to experimentally manipulate information flows. This is a tradeoff between ecological validity and causal identification. Second, one of our goals was to explore the role of Interpersonal communication networks. While one can experimentally manipulate such networks, it is hard to do so in an ecologically valid manner, unless the focus is entirely on weak ties where prior relationships are less crucial.

A consequence of our approach is that we cannot assess whether those inclined to hold the conspiracy beliefs select into networks that are likely to be conspiratorial. This is a crucial question for future work (e.g. Suthaharan and Corlett 2023): whether there are conspiratorial social networks, and if so, what is their nature.

2. Sample Description

The data were collected via an online non-probability sample with quotas for gender, race, age, and region. As mentioned, respondents (N=2,765) were recruited via PureSpectrum between 07/17/2024 and 07/21/2024. The research was reviewed and approved by an Institutional Review Board (IRB STU00219054). In the unweighted data, 62% of the respondents were female, 11% were African American, 3% Asian American, 3% Hispanic, and 3% other race/ethnicity. Additionally, 36% of the respondents were Democrats and 34% were Republicans. The mean age was 57 years (SD=16). Analyses were adjusted using post-stratification weights based on gender, race, age, education, urbanicity, and region, as well as turnout and vote choice in the 2020 presidential election.

3. Question text

4-item American Conspiracy Thinking Scale (ACTS) (Uscinski & Parent 2014).

How much do you agree or disagree with the following statements?

- Even though we live in a democracy, a few people will always run things anyway.
- The people who really 'run' the country are not known to the voters.
- Big events like wars, recessions, and the outcomes of elections, are controlled by small groups of people who are working in secret against the rest of us.
- Much of our lives are being controlled by plots hatched in secret places.

  - Strongly agree
  - Somewhat agree
  - Neither agree nor disagree
  - Somewhat disagree
  - Strongly disagree

How closely do you follow news and information about politics and current affairs?

- Very closely
- Somewhat closely
- Not very closely
- Not closely at all

How would you describe your gender identity?

- Man
- Woman
- Genderqueer/Gender non-conforming
- Another gender identity:___________

What racial or ethnic group best describes you? (Please select all that apply)

- Asian or Asian American
- Black or African American
- Hispanic or Latino
- Native American or Alaska Native
- Pacific Islander or Native Hawaiian
- White or Caucasian
- Other:_____________________________

What is the highest level of education you have completed?

- Grade 9 or less
- Some high school, did not graduate
- High school graduate (diploma, GED, or equivalent)
- Some college, no degree
- Associate degree (AA, AS)
- Bachelor’s degree (BA, BS)
- Graduate degree (master’s, PhD, or professional degree beyond bachelor’s)

What was the total combined income of your household for the past year? Please give us your best estimate.

- Less than $10,000
- $10,000 to $14,999
- $15,000 to $24,999
- $25,000 to $34,999
- $35,000 to $49,999
- $50,000 to $74,999
- $75,000 to $99,999
- $100,000 to $149,999
- $150,000 to $199,999
- $200,000 or more

Sometimes, people can get distracted during a survey. Just so we know you are still with us and paying attention, please select "never" below.

– Always
– Very often
– Sometimes
– Rarely
– Never

In general, how interested are you in US politics and government?

– Extremely interested
– Very interested
– Somewhat interested
– Not very interested
– Not at all interested

Generally speaking, do you think of yourself as a...

– Republican
– Democrat
– Independent
– Other

Did you approve or disapprove of the way Donald Trump handled his job as president?

– Strongly approve
– Approve
– Neither approve or disapprove
– Disapprove
– Strongly disapprove

On Saturday, July 13, a gunman attempted to assassinate Donald Trump at a rally in Philadelphia. Donald Trump was unharmed, but others at the rally were hurt.
Before taking this survey, were you aware of this event?

– Yes
– No

[*If aware of assassination attempt*] Where did you get information about this event? (Please select all that apply)

¾ From people I know
¾ Television
¾ Radio
¾ Newspaper
¾ News website
¾ Social media

¾ Podcast
¾ Other: __________

[*If aware of assassination attempt*] People have heard different things about this event. Have you personally heard or seen any of the following with regard to the attempted assassination of Donald Trump?

- Democratic operatives planned the assassination attempt against Donald Trump
- The assassination attempt against Donald Trump was not real, it was staged

– Yes, I have heard/seen this
– No, I did not hear/see this

[*If heard that the shooting was planned by Democratic operatives*] Where did you hear or see that Democratic operatives were behind the assassination attempt? Please select the sources and type in the name of the specific news outlet or online platform.

¾ From people I know
¾ Television: __________
¾ Radio: __________
¾ Newspaper: __________
¾ News website: __________
¾ Social media: __________
¾ Podcast: __________
¾ Other: __________

[*If heard that the shooting was staged*] Where did you hear or see that the assassination attempt against Donald Trump was staged and not real? Please select the sources and type in the name of the specific news outlet or online platform.

¾ From people I know
¾ Television: __________
¾ Radio: __________
¾ Newspaper: __________
¾ News website: __________
¾ Social media: __________
¾ Podcast: __________
¾ Other: __________

[*If heard that the shooting was planned by Democratic operatives*] How likely do you think it is that Democratic operatives were behind the assassination attempt?

– Very likely
– Somewhat likely
– Neither likely nor unlikely
– Somewhat unlikely
– Very unlikely

[*If heard that the shooting was staged*] How likely do you think it is that the assassination attempt against Donald Trump was staged and not real?

- Very likely
- Somewhat likely
- Neither likely nor unlikely
- Somewhat unlikely
- Very unlikely

4. Descriptive information

The data were collected via an online non-probability sample with quotas for gender, race, age, and region. Respondents (N=2,765) were recruited by the panel sample vendor PureSpectrum between 07/17/2024 and 07/21/2024. The research was reviewed and approved by an Institutional Review Board (IRB STU00219054).
In the unweighted data, 62% of the respondents were female, 11% were African American, 3% Asian American, 3% Hispanic, and 3% other race/ethnicity. Additionally, 36% of the respondents were Democrats and 34% were Republicans. The mean age was 57 years (SD=16). Analyses were adjusted using post-stratification weights based on gender, race, age, education, urbanicity, and region, as well as turnout and vote choice in the 2020 presidential election.

Key variable frequencies:

Aware of the assassination attempt: 94.6%

Heard Democratic operatives planned the assassination attempt against Donald Trump: Yes: 40.8%, No: 59.2%

Heard the assassination attempt against Donald Trump was not real, it was staged: Yes: 53.3%, No: 46.7%

Where did you get information about this event?

- From people I know: 29.9%
- Television: 64.1%
- Radio: 8.8%
- Newspaper: 6.9%
- News website: 27.4%
- Social media: 43.3%
- Podcast: 5.3%
- Other: 3.1%

Where did you hear or see that Democratic operatives were behind the assassination attempt? (Percent from those who had heard about this conspiracy theory)

- People I know: 32.3%
- Television: 27.7%
- Radio: 3.0%
- Newspaper: 2.2%
- News website: 8.3%
- Social media: 52.8%
- Podcast: 4.3%
- Other: 4.7%

Where did you hear or see that the assassination attempt against Donald Trump was staged and not real? (Percent from those who had heard about this conspiracy theory)

- People I know: 34.2%
- Television: 21.4%
- Radio: 1.9%
- Newspaper: 1.0%
- News website: 7.6%
- Social media: 51.7%
- Podcast: 3.4%
- Other: 4.3%

How likely do you think it is that Democratic operatives were behind the assassination attempt? (Percent from those who had heard about this conspiracy theory)

- Very likely: 13.4%
- Somewhat likely: 16.0%
- Neither likely nor unlikely: 21.6%
- Somewhat unlikely: 12.4%
- Very unlikely: 36.7%

How likely do you think it is that the assassination attempt against Donald Trump was staged and not real? (Percent from those who had heard about this conspiracy theory)

- Very likely: 12.0%
- Somewhat likely: 17.1%
- Neither likely nor unlikely: 18.4%
- Somewhat unlikely: 11.3%
- Very unlikely: 41.3%

American Conspiracy Thinking Scale: Range 1-5, M=3.3, SD=1.0

Donald Trump approval: Range 1-5, M= 2.8, SD=1.6

Interest in political news: Range 1-5, M=3.2, SD=1.2

5. Regression results

Table S1. Logistic regression predicting having heard that Democratic operatives planned the assassination attempt against Donald Trump (N=2,543, coefficients are LOR). Media variables record where the respondent learned about the assassination.

Variable	Estimate		Confidence Interval
Male	-0.32	***	[-0.50, -0.14]
Race/Ethnicity: African American	-0.37	*	[-0.68, -0.07]
Race/Ethnicity: Hispanic	-0.13		[-0.53, 0.27]
Race/Ethnicity: Asian American	-0.54	*	[-1.07, -0.03]
Age: 25 to 34	-0.46		[-1.08, 0.15]
Age: 35 to 44	-0.20		[-0.78, 0.38]
Age: 45 to 54	-0.61	*	[-1.18, -0.03]
Age: 55 to 64	-0.54		[-1.11, 0.03]
Age: 65+	-0.51		[-1.08, 0.05]
Education: High School	0.61		[-0.02, 1.29]
Education: Some college	0.67	*	[0.04, 1.36]
Education: College degree	0.59		[-0.05, 1.27]
Education: Graduate degree	0.82	*	[0.14, 1.53]
Income	0.03		[-0.02, 0.07]
Parent	-0.03		[-0.28, 0.21]
Party: Independent	0.03		[-0.20, 0.27]
Party: Other	0.13		[-0.41, 0.65]
Party: Republican	0.11		[-0.18, 0.4]
Trump approval	0.10	*	[0.02, 0.17]
Region: Northwest	-0.10		[-0.37, 0.16]
Region: South	-0.08		[-0.29, 0.13]
Region: West	-0.16		[-0.43, 0.10]
Area: Suburban	-0.25	*	[-0.48, -0.02]
Area: Urban	-0.13		[-0.40, 0.14]
Political interest	0.34	***	[0.23, 0.45]
Conspiracy thinking	0.21	***	[0.12, 0.30]
News: Interpersonal network	-0.01		[-0.20, 0.18]
News: Television	0.12		[-0.08, 0.33]
News: News Website	0.21	*	[0.01, 0.40]
News: Social media	0.60	***	[0.40, 0.79]

* p < .05; ** p <.01; ***p<.001

Table S2. Logistic regression predicting having heard that the assassination attempt against Donald Trump was staged and not real (N=2,538, coefficients are LOR). Media variables record where the respondent learned about the assassination.

Variable	Estimate		Confidence Interval
Male	-0.16		[-0.34, 0.01]
Race/Ethnicity: African American	0.43	**	[0.15, 0.71]
Race/Ethnicity: Hispanic	0.66	***	[0.39, 0.93]
Race/Ethnicity: Asian American	-0.90	***	[-1.28, -0.53]
Age: 25 to 34	-0.31		[-0.67, 0.05]
Age: 35 to 44	-0.29		[-0.65, 0.08]
Age: 45 to 54	-0.60	**	[-0.97, -0.24]
Age: 55 to 64	-0.51	**	[-0.87, -0.15]
Age: 65+	-0.73	***	[-1.09, -0.38]
Education: High School	0.24		[-0.30, 0.78]
Education: Some college	0.27		[-0.28, 0.81]
Education: College degree	0.27		[-0.29, 0.83]
Education: Graduate degree	0.31		[-0.28, 0.91]
Income	0.08	***	[0.04, 0.13]
Parent	-0.14		[-0.36, 0.09]
Party: Independent	0.01		[-0.23, 0.24]
Party: Other	-0.46		[-0.93, 0.010]
Party: Republican	-0.38	**	[-0.67, -0.10]
Trump approval	-0.12	**	[-0.20, -0.05]
Region: Northwest	-0.11		[-0.38, 0.15]
Region: South	0.07		[-0.16, 0.30]
Region: West	0.21		[-0.05, 0.48]
Area: Suburban	0.14		[-0.11, 0.39]
Area: Urban	0.26		[-0.02, 0.55]
Political interest	0.26	***	[0.16, 0.37]
Conspiracy thinking	0.23	***	[0.14, 0.32]
News: Interpersonal network	0.43	***	[0.24, 0.63]
News: Television	-0.04		[-0.23, 0.16]
News: News Website	0.19		[-0.01, 0.39]
News: Social media	0.54	***	[0.36, 0.73]

* p < .05; ** p <.01; ***p<.001

Table S3. OLS regression predicting belief that Democratic operatives planned the assassination attempt against Donald Trump (N=1,050, R^2= .48) among people who were aware of the assassination. Media variables record where the respondent learned about the conspiracy theory.

Variable	Estimate		Confidence Interval
Male	-0.04		[-0.17, 0.09]
Race/Ethnicity: African American	-0.19		[-0.41, 0.03]
Race/Ethnicity: Hispanic	-0.30	**	[-0.50, -0.10]
Race/Ethnicity: Asian American	0.26		[-0.08, 0.61]
Age: 25 to 34	0.06		[-0.19, 0.31]
Age: 35 to 44	-0.30	*	[-0.54, -0.06]
Age: 45 to 54	-0.13		[-0.39, 0.13]
Age: 55 to 64	-0.25		[-0.50, 0.00]
Age: 65+	-0.35	**	[-0.60, -0.10]
Education: High School	-0.25		[-0.67, 0.17]
Education: Some college	-0.06		[-0.48, 0.36]
Education: College degree	0.01		[-0.43, 0.44]
Education: Graduate degree	-0.30		[-0.75, 0.16]
Income	-0.04	*	[-0.08, -0.01]
Parent	0.09		[-0.07, 0.26]
Party: Independent	0.21	*	[0.01, 0.41]
Party: Other	0.43	*	[0.04, 0.82]
Party: Republican	0.16		[-0.07, 0.40]
Trump approval	0.41	***	[0.35, 0.47]
Region: Northwest	0.15		[-0.06, 0.35]
Region: South	-0.09		[-0.27, 0.08]
Region: West	0.05		[-0.15, 0.26]
Area: Suburban	-0.39	***	[-0.57, -0.20]
Area: Urban	-0.20		[-0.41, 0.01]
Political interest	0.03		[-0.05, 0.11]
Conspiracy thinking	0.38	***	[0.31, 0.45]
News: Interpersonal network	0.22	**	[0.06, 0.39]
News: Television	0.21	*	[0.05, 0.38]
News: News Website	-0.06		[-0.30, 0.18]
News: Social media	-0.16	*	[-0.31, -0.01]

* p < .05; ** p <.01; ***p<.001

Table S4. OLS regression predicting belief that the assassination attempt against Donald Trump was staged and not real (N=1,348, R^2= .35) among people who were aware of the assassination. Media variables record where the respondent learned about the conspiracy theory.

Variable	Estimate		Confidence Interval
Male	-0.37	***	[-0.51, -0.24]
Race/Ethnicity: African American	0.37	***	[0.18, 0.56]
Race/Ethnicity: Hispanic	-0.08		[-0.26, 0.10]
Race/Ethnicity: Asian American	-0.02		[-0.36, 0.31]
Age: 25 to 34	-0.64	***	[-0.89, -0.39]
Age: 35 to 44	-0.47	***	[-0.72, -0.21]
Age: 45 to 54	-0.56	***	[-0.82, -0.30]
Age: 55 to 64	-0.68	***	[-0.94, -0.43]
Age: 65+	-0.89	***	[-1.14, -0.63]
Education: High School	0.10		[-0.32, 0.52]
Education: Some college	0.12		[-0.30, 0.55]
Education: College degree	0.17		[-0.26, 0.60]
Education: Graduate degree	0.08		[-0.37, 0.52]
Income	-0.05	**	[-0.08, -0.01]
Parent	0.10		[-0.07, 0.27]
Party: Independent	-0.15		[-0.33, 0.02]
Party: Other	0.03		[-0.35, 0.42]
Party: Republican	-0.54	***	[-0.78, -0.31]
Trump approval	-0.26	***	[-0.31, -0.20]
Region: Northwest	0.09		[-0.12, 0.30]
Region: South	-0.01		[-0.19, 0.17]
Region: West	-0.09		[-0.30, 0.11]
Area: Suburban	0.06		[-0.15, 0.26]
Area: Urban	0.01		[-0.22, 0.24]
Political interest	-0.15	***	[-0.24, -0.07]
Conspiracy thinking	0.49	***	[0.41, 0.56]
News: Interpersonal network	0.37	***	[0.21, 0.53]
News: Television	0.03		[-0.15, 0.20]
News: News Website	0.20		[-0.05, 0.45]
News: Social media	-0.03		[-0.18, 0.13]

* p < .05; ** p <.01; ***p<.001

As an additional robustness check, we examined predictors of believing conspiracy theories among all respondents (regardless of whether they heard the conspiracy or not). Since people who did not report having heard the conspiracy theory were not asked about believing it, their belief was imputed based on demographic and political variables (gender, age, race/ethnicity, income, education, urbanicity, party, and ideology).

We present these results to provide additional information to the readers, but they should be interpreted with a lot of caution. Their findings may not be reliable due to the imputation of belief and the use of less appropriate news source variables.

Table S5. OLS regression predicting belief that Democratic operatives planned the assassination attempt against Donald Trump (N= 2546, R^2=.33) among the full sample. As people who did not hear the conspiracy theory were not asked about belief in it, their score was imputed based on demographics. Media variables record where the respondent learned about the assassination.

Variable	Estimate		Confidence Interval
Male	0.08		[-0.02, 0.18]
Race/Ethnicity: African American	-0.23	**	[-0.38, -0.08]
Race/Ethnicity: Hispanic	-0.24	**	[-0.39, -0.10]
Race/Ethnicity: Asian American	0.26	*	[0.04, 0.47]
Age: 25 to 34	-0.27	**	[-0.46, -0.07]
Age: 35 to 44	-0.34	**	[-0.54, -0.13]
Age: 45 to 54	-0.24	*	[-0.44, -0.03]
Age: 55 to 64	-0.34	***	[-0.55, -0.14]
Age: 65+	-0.47	***	[-0.67, -0.28]
Education: High School	0.20		[-0.10, 0.51]
Education: Some college	0.13		[-0.18, 0.44]
Education: College degree	0.12		[-0.19, 0.44]
Education: Graduate degree	-0.09		[-0.43, 0.25]
Income	-0.06	***	[-0.08, -0.03]
Parent	0.15	*	[0.02, 0.28]
Party: Independent	0.53	***	[0.39, 0.66]
Party: Other	0.90	***	[0.63, 1.17]
Party: Republican	0.83	***	[0.66, 1.00]
Trump approval	0.24	***	[0.20, 0.28]
Region: Northwest	0.08		[-0.08, 0.24]
Region: South	0.10		[-0.03, 0.24]
Region: West	0.18	*	[0.03, 0.33]
Area: Suburban	-0.09		[-0.23, 0.06]
Area: Urban	0.01		[-0.16, 0.17]
Political interest	-0.01		[-0.07, 0.05]
Conspiracy thinking	0.17	***	[0.11, 0.22]
News: Interpersonal network	0.16	**	[0.05, 0.27]
News: Television	0.08		[-0.04, 0.19]
News: News Website	-0.10		[-0.21, 0.02]
News: Social media	-0.01		[-0.12, 0.10]

* $p < .05$; ** $p <.01$; *** $p<.001$

Table S6. OLS regression predicting belief that the assassination attempt against Donald Trump was staged and not real (N=2546, R^2=.20) among the full sample. As people who did not hear the conspiracy theory were not asked about belief in it, their score was imputed based on demographics. Media variables record where the respondent learned about the assassination.

Variable	Estimate		Confidence Interval
Male	-0.29	***	[-0.40, -0.19]
Race/Ethnicity: African American	0.39	***	[0.23, 0.55]
Race/Ethnicity: Hispanic	-0.03		[-0.18, 0.13]
Race/Ethnicity: Asian American	0.09		[-0.14, 0.31]
Age: 25 to 34	-0.39	***	[-0.59, -0.18]
Age: 35 to 44	-0.51	***	[-0.72, -0.30]
Age: 45 to 54	-0.52	***	[-0.74, -0.31]
Age: 55 to 64	-0.68	***	[-0.89, -0.47]
Age: 65+	-0.89	***	[-1.10, -0.68]
Education: High School	-0.07		[-0.38, 0.25]
Education: Some college	-0.17		[-0.49, 0.15]
Education: College degree	-0.19		[-0.52, 0.14]
Education: Graduate degree	-0.08		[-0.43, 0.27]
Income	-0.02		[-0.05, 0.00]
Parent	0.11		[-0.03, 0.24]
Party: Independent	-0.18	*	[-0.32, -0.04]
Party: Other	0.20		[-0.09, 0.48]
Party: Republican	-0.78	***	[-0.95, -0.61]
Trump approval	-0.17	***	[-0.21, -0.13]
Region: Northwest	0.09		[-0.08, 0.25]
Region: South	0.05		[-0.09, 0.19]
Region: West	-0.14		[-0.30, 0.02]
Area: Suburban	0.18	*	[0.03, 0.34]
Area: Urban	0.21	*	[0.04, 0.38]
Political interest	-0.06		[-0.12, 0.00]
Conspiracy thinking	0.21	***	[0.16, 0.27]
News: Interpersonal network	-0.01		[-0.13, 0.10]
News: Television	-0.05		[-0.17, 0.07]
News: News Website	-0.27	***	[-0.39, -0.15]
News: Social media	-0.05		[-0.17, 0.06]

* p < .05; ** p <.01; ***p<.001

6. Supporting Information References

Baker, R., Brick, J.M., Bates, N.A., Battaglia, M., Couper, M.P., Dever, J.A., Gile, K.J., & Tourangeau, R. (2013). Report on the AAPOR Task Force on Non-probability sampling. https://aapor.org/wp-content/uploads/2022/11/NPS_TF_Report_Final_7_revised_FNL_6_22_13-1.pdf

Barthel, M., Mitchell, A., Asare-Marfo, D., Kennedy, D., & Worden, K. (2020). Measuring News Consumption in a Digital Era. Pew Research Center. https://www.pewresearch.org/journalism/2020/12/08/measuring-news-consumption-in-adigital-era/.

Baum, M.A., Druckman, J.N., Simonson, M., Lin, J., & Perlis, R.H. (2024). The Political Consequences of Depression: How Conspiracy Beliefs, Participatory Inclinations, and Depression Relate to Support for Political Violence. *American Journal of Political Science*, *68,* 575-594. https://onlinelibrary.wiley.com/doi/full/10.1111/ajps.12827

Berinsky, A.J. (2023). *Political Rumors*. Princeton University Press.

Bosch, O.J., & Revilla, M. (2022). When Survey Science Met Web Tracking: Presenting an Error Framework for Metered Data. *Journal of the Royal Statistical Society Series A: Statistics in Society*, *185*, S408–S436, https://doi.org/10.1111/rssa.12956

Bradley, V.C., Kuriwaki, S., Isakov, M., Sejdinovic, D., Meng, X. L., & Flaxman, S. (2021). Unrepresentative Big Surveys Significantly Overestimated US Vaccine Uptake. *Nature*, *600*, 695–700. https://doi.org/10.1038/s41586-021-04198-4

Conrad, F.G., Keusch, F. & Schober, M.F. (2021). New Data in Social and Behavioral Research. *Public Opinion Quarterly*, *85*(S1), 253-263.

Dilliplane, S., Goldman, S. K., & Mutz, D. (2013). Televised Exposure to Politics: New Measures for a Fragmented Media Environment. *American Journal of Political Science*, *57*(1), 236–248. https://doi.org/10.1111/j.1540-5907.2012.00600.x

Douglas, K.M., & Sutton, R.M. (2023). What Are Conspiracy Theories? A Definitional Approach to Their Correlates, Consequences, and Communication. *Annual Review of Psychology*, *74*, 271–298. https://doi.org/10.1146/annurev-psych-032420-031329

Druckman, J.N. (2022). *Experimental Thinking: A Primer on Social Science Experiments*. Cambridge University Press

Enns, P.K., & Rothschild, J. (2021). Revisiting the ‘Gold Standard’ of Polling: New Methods Outperformed Traditional Ones in 2020. *3Streams (blog)*. https://medium.com/3streams/revisiting-the-gold-standard-of-polling-new-methods-outperformed-traditional-ones-in-2020-451650a9ba5b

Gelman, A., Goel, S., Rothschild, D., & Wang, W. (2016). High-Frequency Polling with Non-Representative Data. In Schill, D., Kirk, R., & Jasperson, A.E., eds., *Political Communication in Real Time*, Routledge.

Graham, M.H. (2023). Measuring Misperceptions? *American Political Science Review*, *117*(1), 80–102. https://doi.org/10.1017/S0003055422000387

Green, J., Druckman, J.N., Baum, M.A., Ognyanova, K., Simonson, M.D., Perlis, R.H., & Lazer, D. (2023). Media Use and Vaccine Resistance. *PNAS Nexus*, *2*(5). https://doi.org/10.1093/pnasnexus/pgad146

Guess, A., Munger, K., Nagler, J., & Tucker, J. (2019). How Accurate Are Survey Responses on Social Media and Politics? *Political Communication, 36*(2), 241–258. https://doi.org/10.1080/10584609.2018.1504840

Holliday, D., Reny, T., Hayes, A.R., Rudkin, A., Tausanovitch, C., & Vavreck, L. (2021). Democracy Fund + UCLA Nationscape Methodology and Representativeness Assessment. https://www.voterstudygroup.org/data/nationscape

Jungheer, A. (2015). *Analyzing Political Communication with Digital Tarce Data: The Role of Twitter Messages in Science Research*. Springer Publishing Company.

Konitzer, T., Allen, J., Eckman, S., Howland, B., Mobius, M., Rothschild, D., & Watts, D. (2021). Comparing Estimates of News Consumption from Survey and Passively Collected Behavioral Data. *Public Opinion Quarterly*, *85*(S1), 347–370. https://doi.org/10.2139/ssrn.3548690

Lacombe, M.J., Simonson, M.D., Green, J., & Druckman, J.N. (2022). Social Disruption, Gun Buying, and Anti-System Beliefs. *Perspectives on Politics*, 1–18. doi:10.1017/S1537592722003322

MacInnis, B., Krosnick, J. A., Ho, A. S., & Cho, M.-J. (2018). The Accuracy of Measurements with Probability and Nonprobability Survey Samples: Replication and Extension. *Public Opinion Quarterly*, *82*, 707–744. https://doi.org/10.1093/poq/nfy038

Mercer, A., & Lau, A. (2023). Comparing Two Types of Online Survey Samples. Pew Research Center. https://www.pewresearch.org/methods/2023/09/07/comparing-two-types-of-online-survey-samples/.

Nyhan, B., Settle, J., Thorson, E. et al. (2023). Like-minded Sources on Facebook are Prevalent But Not Polarizing. *Nature*, *620*, 137–144. https://doi.org/10.1038/s41586-023-06297-w

Parry, D.A., Davidson, B.I., Sewall, C.J.R., Fisher, J.T., Mieczkowski, H., & Quintana, D.S. (2021). A Systematic Review and Meta-Analysis of Discrepancies between Logged

and Self-Reported Digital Media Use. *Nature Human Behaviour*, *5*(11), 1535–1547. https://doi.org/10.1038/s41562-021-01117-5

Quintana-Mathe, A., Uslu, A.A., Radford, J., Druckman, J.N., Lunz Trujillo, K., Safarpour, A., Ognyanova, K., Baum, M.A., Schulman, J., Perlis, R.H.., Santillana, M., Lazer, David. (2024). Large, Non-Probability Surveys Can Produce Valid Estimates of COVID-19 Vaccination and Cumulative Infection Rates. OSF Preprints. https://osf.io/4hgyr.

Radford, J., Green, J., Quintana, A., Safarpour, A., Simonson, M. D., Baum, M., Lazer, D., et al. (2022). Evaluating the Generalizability of the COVID States Survey — a Large-Scale, Non-Probability Survey. OSF Preprints. https://doi.org/10.31219/osf.io/cwkg7

Santillana M., Uslu A.A., Urmi T., Quintana-Mathe, A., Druckman, J.N., Ognyanova, K., Baum, M., Perlis, R.H., & Lazer, D. (2024). Tracking COVID-19 Infections Using Survey Data on Rapid At-Home Tests. *JAMA Network Open*, *7*(9), e2435442. doi:10.1001/jamanetworkopen.2024.35442

Scharkow, M. (2016). The Accuracy of Self-Reported Internet Use—A Validation Study Using Client Log Data. *Communication Methods and Measures*, *10*(1), 13–27. https://doi.org/10.1080/19312458.2015.1118446

Settle, J. (2018). *Frenemies: How Social Media Polarizes America*. Cambridge University Press.

Simonson, M.D., Block Jr., R., Druckman, J.N., Ognyanova, K., & Lazer, D. (2024). *Black Networks Matter: The Role of Interracial Contact and Social Media in the 2020 Black Lives Matter Protests.* Cambridge University Press. https://www.cambridge.org/core/elements/black-networks-matter/F1D7CC20FE798260F22D3E40ABE26A03

Suthaharan, P., & Corlett, P.R. (2023). Assumed Shared Belief about Conspiracy Theories in Social Networks Protects Paranoid Individuals Against Distress. *Scientific Reports*, *13*, 6084. https://doi.org/10.1038/s41598-023-33305-w

Tourangeau, R., Rips, L. J., & Rasinski, K. (2000). *The Psychology of Survey Response.* Cambridge University Press. https://doi.org/10.1017/CBO9780511819322

Uscinski, J. E., & Parent, J. M. (2014). *American Conspiracy Theories*. Oxford University Press.

Vehovar, V., Toepoel, V., & Steinmetz, S. (2016). Non-probability Sampling. In Wolf, C., Joy, D., Smith, T.W., & Fu, Y., eds. *The Sage Handbook of Survey Methodology*. Sage.
